# Advances on the Valorisation and Functionalization of By-Products and Wastes from Cereal-Based Processing Industry

**DOI:** 10.3390/foods9091243

**Published:** 2020-09-05

**Authors:** Adriana Skendi, Kyriaki G. Zinoviadou, Maria Papageorgiou, João M. Rocha

**Affiliations:** 1 Department of Food Science and Technology, International Hellenic University, P.O. Box 141, GR-57400 Thessaloniki, Greece; andrianaskendi@hotmail.com; 2 Department of Food Science and Technology, Perrotis College, American Farm School, GR-57001 Thessaloniki, Greece; kzinov@afs.edu.gr; 3 REQUIMTE—Chemistry and Technology Network, Green Chemistry Laboratory (LAQV), Department of Chemistry and Biochemistry, Faculty of Sciences—University of Porto (FCUP), Rua do Campo Alegre, s/n., P-4169-007 Porto, Portugal; jrocha@sourdomics.com or

**Keywords:** cereal and by-products, arabinoxylans, β-glucans, dietary fibres, biopolymer-based packaging, new product development (NPD)

## Abstract

Cereals have been one of the major food resources for human diets and animal feed for thousands of years, and a large quantity of by-products is generated throughout the entire processing food chain, from farm to fork. These by-products mostly consist of the germ and outer layers (bran) derived from dry and wet milling of the grains, of the brewers’ spent grain generated in the brewing industry, or comprise other types obtained from the breadmaking and starch production industries. Cereal processing by-products are an excellent low-cost source of various compounds such as dietary fibres, proteins, carbohydrates and sugars, minerals and antioxidants (such as polyphenols and vitamins), among others. Often, they are downgraded and end up as waste or, in the best case, are used as animal feed or fertilizers. With the increase in world population coupled with the growing awareness about environmental sustainability and healthy life-styles and well-being, the interest of the industry and the global market to provide novel, sustainable and innovative solutions for the management of cereal-based by-products is also growing rapidly. In that respect, these promising materials can be valorised by applying various biotechnological techniques, thus leading to numerous economic and environmental advantages as well as important opportunities towards new product development (NPD) in the food and feed industry and other types such as chemical, packaging, nutraceutical (dietary supplements and food additives), cosmetic and pharmaceutical industries. This review aims at giving a scientific overview of the potential and the latest advances on the valorisation of cereal-based by-products and wastes. We intended it to be a reference document for scientists, technicians and all those chasing new research topics and opportunities to explore cereal-based by-products through a circular economy approach.

## 1. Introduction

A society without hunger but with quality and safe food for all is one of the largest challenges for humanity. It has been calculated that 1.3 billion tons of food loss and waste are generated every year from all stages of the food manufacturing process, chiefly food production and consumption [1]. The latter corresponds to approximately one-third of the food produced in the world for human consumption that is lost or becomes waste every year [2]. Paradoxically, an impressive one-third of the world’s soil has been lost in the last 40 years, 2.3 billion square kilometres of land area are being degraded and almost 5 million of people died from hunger just in 2020 [2].

These losses result from the whole food (and feed) value chain, i.e., from the harvest step, passing through the postharvest processing, industrial processing and commercialization, to the final consumption by the consumers (transportation, storage, home processing and waste). Food losses also include food processing by-products, which remain as side streams during the production of target products among all sectors of food production. Of course, the negative impact of these losses is even greater if the required inputs such as land, energy and water resources are taken into consideration. This scenario has raised a global interest and the Zero Hunger and the Responsible Consumption and Production of food plans have been included in the seventeen Global Sustainability Goals of the United Nations (UN), which represent an Agenda for global action up to 2030 and defines the sustainable development at the economic, social and environmental dimensions [3]. The “*Farm to Fork Strategy—for a fair, healthy and environmentally-friendly food system*” [4] is also a good example of the European Union (EU) set of strategies and actions on the scope of “*The European Green Deal*” towards the EU’s economy sustainable [5]. These policies are in line with the strategies and commitments of the Food and Agriculture Organization (FAO) of the United Nations towards a sustainable future.

Over decades, the EU has developed and put into force some world’s highest environmental standards, dating back from 2008 when the EU waste management law established a legal framework to treat wastes [6]. The directive 2008/98/EC [7] is of major importance since it gave priority to the reduction of the waste at the source, followed by reuse, recycling, and recovery, i.e., the 3 R’s policy following the hierarchy: Reduce, Reuse and Recycle. The 3 R’s policy is often replaced by the 5 R’s concept, i.e., Reduce, Reuse, Recycle, Rethink and Renew. In that respect, the utilization of by-products from Agro-food manufacturing industry affects industrial branches with underestimated capacity and growing amounts of by-products, against the trends of sustainability principles [8].

The term “cereals” refers to members of the Gramineae family and determines nine species: wheat (*Triticum* spp.), rye (*Secale* spp.), barley (*Hordeum* spp.), oat (*Avena* spp.), rice (*Oryza* spp.), millet (*Pennisetum* spp.), corn (*Zea* spp.), sorghum (*Sorghum* spp.), and triticale (x *Triticosecale* Wittmack)—which is a hybrid of wheat and rye. Furthermore, pseudocereals are non-grasses with similar end-uses to cereals (or true grasses); these flours have gained high popularity among scientists, technicians, and consumers, and encompass the well-known buckwheat (*Fagopyrum esculentum*), amaranth (*Amaranthus* spp.), quinoa (*Chenopodium* spp.), and others such as chia (*Salvia* spp.), *cañihua* and pit seed goosefoot (*Chenopodium* spp.), breadnut (*Brosimum alicastrum*), celosia (*Celosia* spp.) and wattle seeds (*Acacia* spp.).

**Figure 1:**
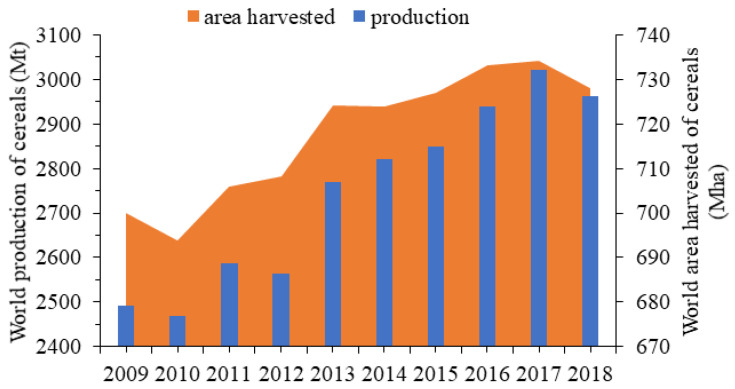
World cereal harvested area and production during the time period of 2009–2018. Data source: FAOSTAT [9].

For a majority of the world’s population, cereals represent a staple food in their daily diet. As depicted in Figure 1, a continuous increase—both in cereal production and corresponding harvested area—is being perceived and can be mainly attributed to the growing demands due to the global human’s demographic growth or human overpopulation, especially after World War II. Particularly during 2018, an increase of 4.0% in the globally harvested area and 18.9% in the cereal production occurred compared to 2009.

**Figure 2:**
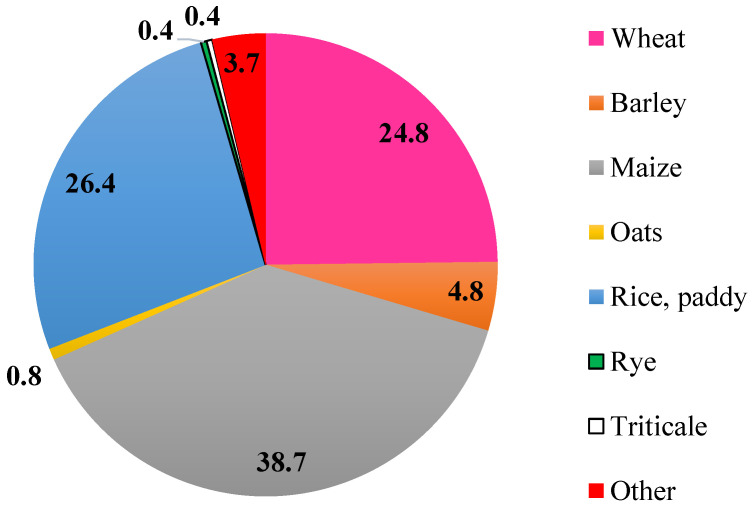
Percentage of world cereals production during 2018. Data source: FAOSTAT [9].

**Figure 3:**
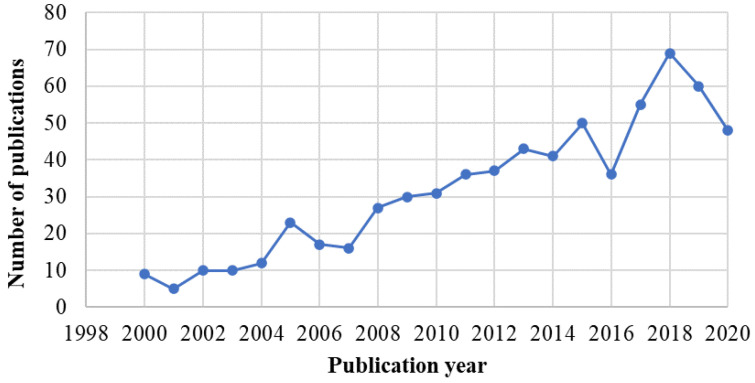
Number of yearly articles published on the topic of cereal by-products during the period 1920–2020, according to the Scopus database (https://www.scopus.com/home.uri). Last access on 09/07/2020; Document search: “*cereal AND by-product*”.

The three main cereals produced in the world during 2018 were maize (38.7%), rice (26.4%) and wheat (24.8%), comprising 89.9% of the world’s production of cereals (Figure 2). Despite being rich in nutrients, cereal by-products are still mostly being turned out in feed, substrates for bio-refineries or waste. Cereal processing industry is striving to find alternative ways to minimize their volumes of by-products and wastes. In that way, they are looking for new solutions and applications to valorise their by-products by the means of their reprocessing in order to get valuable extractable compounds presented therein or chemically, microbiologically and enzymatically converted compounds, so that new applications and niches of markets are attained. In response to that, the scientific literature reports a significant growing number of publications during the last twenty years dealing with cereal by-products (Figure 3).

In cereal grains, the carbohydrates are the major constituents and encompass starch and soluble sugars as well as carbohydrates not digested by humans, i.e., the dietary fibres: hemicellulose (β-glucans and arabinoxylans), cellulose, lignin, pectin, resistant starch and other complex polysaccharides. Starch is the most abundant cereal polysaccharide, contributing to ca. 40–70%. Dietary fibres include, for instance, the water-soluble β-glucan and water-soluble pentosans, whereas the water-insoluble fibres are the lignin, cellulose, water-insoluble hemicelluloses [10]. Extractable plant-based components include fibres, hemicelluloses, proteins, polysaccharides, lipids and phytochemicals. Proteins comprise 7–12% of cereals whereas lipids are minor compounds comprising ca. 1.7–5.7% (depending on the type of cereal and flour extraction rates) and are mainly formed by neutral, glyco- and phospholipids [11,12,13,14,15,16,17,18,19,20].

We intend this scientific review article to give an original overview of the high value of cereal-based by-products and their processing derivatives. Based on the identification of their most valuable constituents, numerous innovative technological routes were identified and applied to virtually most industrial segments. Furthermore, the use of cereal-based by-products was treated in this research work not only from a scientific-technological point of view but rather in an integrated perspective of environmental, economic and social sustainability. It is anticipated that this review article may become a reference document for those scientists and technicians who need new insights on the topic of cereal-based by-product valorisation.

## 2. Processing of Cereals and the Derived By-Products

A detailed description of cereal processing and the resulting by-products can be obtained in some complementary literature [21,22,23]. In the present section, a brief description of the various alternatives for cereal processing will be presented and will include the most recent advances in the field.

Cereal by-products continue to represent an unexploited source of many compounds or fractions with high nutritional value and could serve as novel material not only for food and feed production but also for other non-food products. Besides the food industry, bioethanol industry represents an important alternative for the use of cereal by-products.

The milling industry is the major supplier of cereal by-products. The steps performed before milling result in a by-product called “grain screenings” mainly containing all the cereal seeds that do not fulfil grading specification. Besides the refined cereal flours, cereal bran and germ are separately obtained during milling, with the latter used subsequently for the production of cereal germ oil. Cereal bran is considered the main by-product from cereal milling. It contains the coat seed and aleurone layer from the milling of the cereal grains after sieving out the endosperm. Milling of cereals comprises steps such as cleaning and grading before breaking down the grains and sifting to obtain different fractions based on size-exclusion separation in sieves. Depending on the mill used, the cereal grains are broken into smaller pieces by grinding, cutting or crushing to any desired size specification, depending on the final use. Accordingly, different by-products are produced during cereal processing. According to Ma et al. [24], and as expected, flours with smaller particle size had higher amounts of damaged starch but also lower levels of sulfhydryl content of gluten protein leading to alteration of the quality characteristics of the flours. Depending on the desired traits of the final product (i.e., refined flour, starch and gluten), appropriate milling processes and equipment may be applied, e.g., jet milling combined with successive grindings in cascade and air classification are employed to isolate starch from flours resulting in a final product containing <2% (dry basis, d.b.) of residual protein and very low amounts of lipids and pentosans [25].

Besides the refined wheat flours, nowadays the market requests the production of other types of flours such as wholemeal or enriched/biofortified flours with plant-based products. For whole wheat flour production, the wheat kernels are ground without separating the endosperm from germ and bran. Even though the consumers’ awareness and acceptance for the consumption of whole grain products is increasing, the industry still needs to focus on the optimization of such products due to the high number of whole grain-avoiders on the cause of poor organoleptic characteristics [26]. Different techniques such as physical, chemical and enzymatic have been applied to this purpose. Besides the increasing of the nutritional value of the final products, these methods aim at reducing negative effects as well as at improving food texture and sensory attributes. Wheat protein that represents a by-product from the industrial production of starch, finds an end-use in the bakery industry to enrich flours for bread and pasta.

Bran from rice, obtained after polishing brown rice, contains grain parts such as pericarp, aleurone and embryo. It is considered as having prebiotic potential because of the beneficial compounds presented therein, such as dietary fibres, essential fatty acids, polyphenols and antioxidants. Bran from rice must be stabilized before use to avoid lipid oxidation and deterioration of its quality and organoleptic characteristics. Recently it has been used in (micro/nano) encapsulate matrices for probiotics showing high encapsulation efficiency and enhanced protection to simulated gastrointestinal tract and storage conditions [27,28]. In addition, it was successfully added in yoghurts in order to increase its antioxidant activity, causing a decrease in syneresis and viscosity values [29]. Further treatment of rice bran can result in the extraction of valuable bioactive compounds such as phenolic compounds. Enzymatic treatments can be alternatively used to minimize the negative effects of traditional extraction methods (chemical degradation or disruption of plant cell wall matrices by acid or base hydrolyses) on the extractability and antioxidant activity of the phenolics. Kim and Lim [30] observed that commercially available carbohydrate enzymes increased the efficiency of phenolic acid extraction from rice bran suggesting the use of this enzymatic treatment to improve the antioxidant activity of the rice bran. In addition to the cereal bran, their oils and cakes are also considered cereal by-products. In the case of rice bran oil, the main components are unsaturated fatty acids (mainly oleic and linoleic acids), and phytochemicals such as phytosterols, vitamin E and γ-oryzanol, which have significant antioxidant activity and important role in the inhibition of some chronic diseases [30]. In terms of the nutritional value of cereal lipids, the importance is well recognized of the relative high content of polyunsaturated fatty acids (PUFA) with different chain lengths, present in free form (free fatty acids) and the acylated forms of triacylglycerols (TAG), diacylglycerols (DAG) and, in relative minor amounts, the monoacylglycerols (MAG). As DAG and MAG, the presence of polyunsaturated sterol esters (SE), as well as sterols and stanols, reveals the high nutritional value of cereals [12,14,15,16,17,18,19,20]. The extraction methods employed affect the quality of the resulting oil. Compared to the conventional methods, the environment-friendly supercritical carbon dioxide extraction (SC-CO_2_) results in better quality bran oils with better physicochemical and antioxidant properties [31]. Rice bran oil can be used to enrich diets since it provides immune enhancement along with other bioactive properties [32]. From the industrial production of starch, rice protein is derived that is not considered for food purposes but only for animal feed [33].

Maize is processed by using two different milling techniques (dry and wet milling), depending on the desired product, giving rise to the production of maize bran/fibre. The dry milling method is employed to obtain the maize endosperm fraction to be used as meals, flours, or grits, resulting also in the production of maize bran fraction (from maize pericarp), while the maize germ remains with the latter, representing a by-product that can be used for the oil production. Besides bran, the cake from maize is considered a residual cereal by-product, obtained from the extraction of oil during the process of de-oiling maize germ. Aqueous extract of maize cake, rich in dietary fibres, that remains after obtaining maize oil, has been utilized for the fortification of cakes with edible fibres to produce health-promoting food products [34]. On the other hand, during wet milling of maize, the grain is first steeped in water and sulphur dioxide to soften the kernels and facilitate the separation of the desired products: starch and germ (for oil extraction). Wet milling of maize results in by-products such as maize fibre from the pericarp and maize protein, while various solids are obtained during steeping. The milling industry is facing with increasing amounts of maize bran obtained by the dry milling process of maize because of the growing demand of maize flour for the bakery industry. This by-product represents a potential source of polymers (typically composed by 50% heteroxylans and 20% cellulose) with high added value for the food industry. In addition, it contains ca. 4% phenolic acids, mainly ferulic and diferulic acids [35]. Further extraction of heteroxylans from maize bran results in water-soluble maize bran gum, another by-product for the use in the food industry [36]. In addition to numerous technological applications in the food, nutraceutical and pharmaceutical industries, maize bran gum can be used as a carrier of bioactive compounds in the intestine. It has the ability to form hydrogels that have the potential for transferring thermosensitive bioactive compounds in the food industry [37]. Besides the milling industry, maize is used as the largest source for bioethanol production and other renewable biofuels such as biodiesel and biogas. One of the significant by-products is the fraction of dried distiller’s grain with solubles (DDGS). They are used as a raw material for the extraction of arabinoxylans (AXs) [38]. Theoretically, maize bran and fibres consist of the pericarp coating but in the case of maize fibres for starch production, maize pericarp is usually mixed with cell wall materials from the maize endosperm, whereas, during ethanol production, maize bran is mixed with soluble fractions from distiller’s grains [39].

Milling and malt-based alcoholic beverage (mainly brewer’s grains) by-products are the main residual products from the barley processing industry. Only hull-less barley varieties are used for food purposes, whereas hulled barley is preferred for malting. Fine flour and course meal are obtained as milling by-products of pearled barley [39,40] whereas the spent grains are the main by-product from brewing. The spent grains have been used as a substrate for immobilization of different enzymes such as trypsin, lipases and laccases [41,42].

Bran from rye and triticale is considered as a by-product obtained after milling. Oat bran is also produced during milling to obtain flours for human consumption. It contains mainly hulls and residues from other processes performed also on the oats such as dehulling, rolling and flaking [40]. In general, endosperm moieties are found in cereal bran of these grains depending on the efficiency of the milling process.

In general, bran is used for the biofortification of refined wheat flour used for breadmaking and other bakery goods. Bread biofortification with bran or whole meal of maize, rice, oat, rye, barley and wheat results in technological changes such as the decrease in loaf bread volume, while the content of water, mineral content, fibres, phytates and antioxidant activity are increased and, in addition, the quality of volatile compounds is also affected [43,44,45,46]. The presence of bran in bakery is linked with increased water absorption and low loaf volume due to the weakening of the dough structure from the disruption of the gluten network. Bran processing is likely to be a route to optimize its incorporation in health-promoting foods with biological activity and/or technological functionalities. For instance, it was reported the enhancement of bread quality by lowering the whole wheat flour particle size [47]. As an alternative to the conventional milling processes referred earlier, recent processes such as microfluidization and micron technology are used to reduce the particle size. The microfluidization process is used to reduce the size of the bran particles to submicron scales and reorganize the fibre composition, as well as to open their dense microstructure, increasing health-beneficial properties such as hydration properties and bio-accessibility of bound phytochemicals, resulting in increased antioxidant activities [48,49]. Ortiz de Erive et al. [50] have successfully produced high-fibre bread with similar loaf volume to the control bread by replacing 20% of the wheat flour with microfluidized corn bran. Similar to the microfluidization process, micron technology is used to reduce the particle size of biomaterials and, consequently, to increase the level of released bioactive compounds. As a matter of fact, micron technology improves the functional properties and bio-accessibility of dietary fibres and other bioactive compounds [51]. In addition to the bakery, wheat bran can be used to yield functional nanomaterials from the derived polysaccharide arabinoxylan (AX). The chemically modified AX can be used as a functional nanomaterial for gene delivery [52] or to produce physically immobilized xylanases [42].

## 3. Dietary Fibres Extracted from Cereal Processing By-Products

Many studies found in the literature report the beneficial effects of dietary fibres against several chronic diseases and the contribution towards the promotion of human or animal health benefits [11,53,54]. The dietary fibres are recognized to contribute to the reduction of glycaemic response, regulation of blood lipids, and an increase in the antioxidant activity, favour weight loss, and promote favourable microbiota in the small intestine and colon [55]. The beneficial effects of dietary fibres were demonstrated in experiments performed in vitro, as well as in vivo in animal and human trials and led to the official recognition of the positive nutritional effects by the U.S. Food and Drug Administration (FDA) [56] and the EU [57]. In the European Union, food is considered as a “*source of fibre*” and “*high content in fibre*” if it provides an amount of fibre equal or above 3 and 6 g per 100 g of product, respectively [58]. According to the European Food Safety Authority (EFSA), whole grain cereals represent a major dietary source of dietary fibres [59]. Recent studies confirmed that cereal fibre intake was inversely associated with cardiovascular disease (CVD) risk factors [60], body mass index (BMI) and waist circumference and tended to be inversely associated with total and low-density lipoprotein (LDL) cholesterol [61] and type-*2* diabetes [62]. In cereals and pseudocereals, dietary fibres contain different compounds, mostly found in the outer layers of the grains and cell walls. These compounds are polymers such as cellulose, lignin, arabinoxylans and (1-3) (1-4)-β-glucans (referred as β-glucans), which are slowly digested or not digested in the human gastrointestinal tract. Alongside the prebiotic effect, some of these polymers have the ability to enhance the absorption of minerals, to lower the cholesterol levels and to slow down starch hydrolysis, thus improving blood sugar levels by flattening of postprandial blood glucose and insulin rise, to activate the immune responses resulting in a reduction of the risk of type-*2* diabetes, CVD’s and colorectal cancers [63]. The health-promoting properties associated with dietary fibres resulted in an increased offer of fibre-rich products in the market.

Different dietary fibres lead to different properties, such as particle size, swelling and water retention capacity, water solubility and viscosity with direct impact on nutritional and health effects and, very importantly, in technological properties of food, such as texture, rheology and sensory features. Dietary fibres are divided into water-soluble and water-insoluble fibres. The latter are structural or non-viscous fibres such as lignin, cellulose, hemicelluloses and non-starch polysaccharides which also include some arabinoxylans not extractable by water. Vegetables and cereal grains are particularly rich in water-insoluble fibres, with the highest amounts found in wheat and corn. Unlike the latter, water-soluble dietary fibres are gel-forming or viscous compounds that encompass β-glucans, arabinoxylans, pectins, gums, mucilage and some hemicelluloses [64].

The most recognized water-soluble dietary fibres associated with health claims are the β-glucans and arabinoxylans. Beta-glucans are made of a linear chain of β-d-glucopyranosyl units linked by β-(1→4) and β-(121923) linkages. The β-(1→3) linkages, that occur alone, break-up the regular/linear cellulose-like type structure formed by up to 11 continuous β-d-glucopyranosyl units linked with β-(1→4) linkages, increasing their solubility in water and limiting chain interactions with other β-glucan chains. Over 90% of the polysaccharide chain comprises cellotriosyl (3 degrees of polymerization, DP3) and cellotetraosyl (DP4) units linked through β-(1→3) linkages [65]. Although present in all cereals, the highest levels of β-glucans are observed in oats (3–7%) and barley (3–11%) [66]. Their amount depends on a number of factors such as genetic, varietal, environmental, growing location, soil composition, etc. Moreover, β-glucans are found in greater amounts in oat bran fractions (sub-aleurone and aleurone layers), whereas in barley grains it is evenly distributed throughout the aleurone layer and endosperm [67,68]. The molecular weight of cereal β-glucans varies in a relatively wide range (from 20 to 3100 kDa) and depends on the botanical origin, genotype and agronomic factors as well as on the extraction protocols applied [65,69,70,71,72]. Higher molecular weight β-glucans present higher viscosity. A study revealed that the increase in β-glucan’s viscosity is associated with positive effects on postprandial glucose, insulin and gastric emptying [73]. Besides the molecular weight, chemical structural differences in β-glucans affect their physiochemical properties such as solubility, swelling and gelatinization (gel formation) capacity, as well as viscosity and water-binding properties. The ratio of DP3 to DP4 linkages and the presence of long cellulose-like fragments with high degrees of polymerization are also key-features when studying β-glucans [65,71].

It is accepted that beta-glucans lower the glycaemic index, blood sugar and LDL cholesterol, as well having antioxidant, anticancer and free radical scavenging properties [74]. It is believed that they play prophylactic roles against colorectal cancer due to their contribution to the increase in faecal and colon mass as a result of the enhanced resistance to digestion. Other effects of β-glucans also include diminished absorption of nutrients, prolonged postprandial satiety and increased stool bulk and relief of constipation. Regarding the prebiotic effects, water-soluble fibres are substrates for the development of desirable colonic microbiota with probiotic features which, in turn, lead to the production of several bioactive postbiotics, such as exopolysaccharides (EPS, e.g., dextrans, levans, fructans and reuterans), and to the growth of deleterious microorganisms. A healthy gut microbiome is indeed crucial for a good immune system and nutrient metabolism, and to the concomitant prevention of several diseases [54,75,76]. Using pure preparations of β-glucans as a food ingredient has been limited because of the involved processing costs but the use of cereal-based by-products, the use of bulk fractions (instead of pure compounds) and the use of novel separation and purification processes may bring cost-effective solutions.

Arabinoxylans (AXs) are hemicelluloses from the primary and secondary plant cell walls, including woods and cereal grains and entailing co-polymers of two pentose sugars, arabinose and xylose [77]. AXs occur in percentages of ca. 1.5 to 2.5% in wheat flour and are considered the predominant cell wall polysaccharides in rye (8–12.1% of the whole grain) [78,79] and wheat (4.1–9%) [79] but they are also present in rice bran [80]. AXs are made of a linear chain backbone of β-D-xylopyranosyl (Xylp) units linked with (1→4) glycosidic bonds. Some of these units are linked by the oxygen (O) in O2, O3 or both positions simultaneously from α-L-arabinofuranosyl (Araf) units. Some Araf units are covalently linked with ferulic acid (FA) through an ester linkage at the C5 position [81,82]. Besides the molecular weight, the amount of attached Araf units as well as the degree of substitution (arabinose to xylose ratio, A/X) affects the physicochemical properties of arabinoxylans [79,83]. Interaction with other AX chains or other compounds within the cereal matrix through ferulic bounds affect the solubility and availability of these polysaccharides, factors connected to their biological activities and health benefits. Based on their solubility and extractability with water, arabinoxylans are categorized into water-extractable (WEAX) and water-unextractable (WUAX). Water extractable arabinoxylans are a minor constituent of AXs but with major influence in the technological and health effects. The molecular weight and AX structure vary according to the botanical origin of the cereals, the cultivars, the agronomic parameters as well with the part of the seed. In rice and maize, the structure is more complex than that found in wheat and rye grains since it contains xylopyranose, galactopyranose and glucuronic acid [36,84]. Moreover, the amount of AXs is higher in the bran fraction than in the starchy endosperm of the cereals [82]. The health beneficial effects of β-glucans and arabinoxylans from cereals make them valuable components of dietary fibres. Solubility, high water adsorption capacity and (as previously said) the ability to increase the viscosity and/or form gel in β-glucans and arabinoxylans depend on the molecular weight and structure of these polysaccharides [65,81,83], modulating their specific health promoting (and functional) effects. Just as an example, WEAX can protect against free radicals in food matrixes and mammalian’s gastrointestinal tract [85].

Maize bran can be used to obtain water-soluble maize bran gum (alkali-extracted under mild conditions) with high arabinoxylan (74% *w/w*) content with an A/X ratio of 0.85 indicating a moderated branched structure [36]. In addition, maize bran gum contains glucose (5.1%), galactose (3.2%) and mannose (0.4%). In order to avoid negative effects on human health from the use of chemicals used for the extraction of the AXs present in maize bran, water can replace the alkaline medium, thus making their extraction more affordable and less toxic [80].

Processing of cereals such as milling and extrusion are considered responsible for the AX degradation—resulting in structural modifications with consequences in its functionality and health promoting effects [78,86]. Milling reduces the molecular weight of insoluble arabinoxylans with high molecular weight and increases significantly their solubility [86]. On the other hand, extrusion affects the AX fine structure and solubility of cereal dietary fibres [80,86].

It was hypothesized that extrusion cooking increases dietary fibre solubility, probably due to the reduction in particle sizes of extruded samples [87] and/or the breaking down of covalent and non-covalent bonds in larger molecules due to the high temperature applied [88]. It was reported that extrusion of rice bran increases the extractable levels of AXs but decreases the A/X ratio and molecular weight compared with non-extruded samples [80,89]. Such an effect is likely to be owing to the high shear-stress forces and high temperatures applied that can cause the release of ferulic acid, softening of lignin and AXs depolymerization. In other studies it was demonstrated that modification of extrusion conditions resulted in an increase in the WEAX and ferulic acid yields from corn and wheat bran [90,91].

Cereal arabinoxylan-oligosaccharides (AXOS) derived from arabinoxylans are generally acknowledged for their prebiotic potential in the colon of humans and animals [92,93]. Obtaining AXOS by enzymatic processes is of particular interest when producing functional foods since they are considered free of chemicals. Usually enzymes such as cellulases, β-glucanases and xylanases are involved in the solubilisation of arabinoxylans in wheat bran, aleurone fraction and outer pericarp fraction so that high yields are achieved [94]. Vangsøe, Sørensen and Bach Knudsen [94] noticed a substantial contribution of aleurone fraction to the production of AXOS in wheat bran. It was observed that among the structural features of the arabinoxylans, the A/X ratio affects the yield of AXOS [94]. On the other hand, modification of wheat bran particle size and tissue composition affects their prebiotic potential. Besides arabinoxylans and β-glucans in cereals, other polysaccharides can be found such as arabinogalactans. Wheat bran is richer in arabinogalactans than the respective whole flour (1.07–4.43% vs. 0.47–0.93%, respectively) [95].

Hydrolysis of residual lignocelluloses or starch yield fermentable sugars could be further used in multiple applications, e.g., single cell proteins (SCP) and oils (SCO); renewable biofuels, from bacteria, yeasts, moulds (*Filamentous fungi*) and microalgae; biofertilizers; chemicals; enzymes; [96,97,98,99,100,101] or microbial metabolites, such as fatty acids, bioactive exopolysaccharides and peptides, amino acids, pigments, organic acids and many other antioxidants and antimicrobials [53,54,75,76]. Analysing the most recent published literature, different techniques depicted in Table 1 were found to facilitate the extraction of non-starch polysaccharides in cereals.

## 4. Phytochemicals Extracted from Cereal Processing By-Products

Cereal by-products are important sources of various phytochemicals. As stated by Wang et al. [137] rice bran contains high levels of various phytochemicals, such as γ-oryzanol, phytic acid, tocopherols, tocotrienols, carotenoids, γ-aminobutyric acid (GABA), octacosanol, squalene, mono- (MUFA) and polyunsaturated fatty acids (PUFA), phytosterols and phenolic compounds [12,13,14,15,16]. From the above list, phenolics have gained major attention lately from scientists, nutritionists and technicians since they exhibit a diverse range of functional abilities (biological activities and/or technological functionalities), such as antioxidant, antimicrobial, antiviral and anti-inflammatory properties [53,54,76]. In recent years, most attempts for the extraction and recovery of phenolic compounds from cereal by-products were based on conventional techniques of solvent extraction resorting to organic ones such as methanol, ethanol, propanol, acetone, ethylacetate, dimethylformamide and/or their combinations. These extraction methods have, however, several drawbacks since they are time-consuming, present low selectivity and low extraction rates and, most importantly, require the use of expensive, volatile and, sometimes, toxic solvents [138]. Recent works dealing with alternative methods for the extraction of phenolic compounds are summarized in Table 2.

The application of the environmentally friendly subcritical water extraction (SWE, i.e., extraction with water under pressure at temperatures above the boiling point at atmospheric temperature and at the sea level) for the hydrolysis and decomposition of rice bran has been applied in order to obtain phenolic compounds and other material of commercial interest. Research findings demonstrated the positive effect of temperature on the total phenolic content and antioxidant activity, while residence-time up to 15 min seemed to be the optimum conditions [149]. Recently, the effect of thermal processing (80 °C for 10 min), coupled with ultrasound-assisted extraction on the antioxidant, antimutagenic and antimicrobial activities of wheat and oat bran was assessed. It was demonstrated that the thermal process improved the total phenolic content in wheat bran by +22.5% and in oat bran by +25.8% [143]. Likewise, the effective extrusion conditions to achieve the highest content of phenolic compounds and antioxidant activity in wheat bran were studied using response surface methodology, and the results were very promising as well [150].

Anthocyanins (a type of flavonoid) from black rice bran have also been isolated and characterized, and recently they have been incorporated into gelatine or chitosan nanocomposite films containing oxidized chitin nanocrystals for fish and seafood spoilage monitoring. In both cases the developed films were pH-sensitive and showed remarkable colour changes in buffer solutions, therefore allowing them to be used as smart food packaging [151,152]. Last but not least, it was found that up to 88% of all extracts in maize fibres and up to 95% in rice husks are lipophilic compounds, i.e., n-fatty acids and acylglycerols (i.e., mono-, di- and triglycerides), accounting for 4.1% and 2.2% of their respective weight and making from these by-products an inexpensive source of valuable phytochemicals [17].

## 5. Proteins Extracted from Cereal Processing By-Products

It is widely known that the supply and demand of proteins for human consumption is going to be subject to a serious imbalance in the near future. There is an increasing trend towards the consumption of plant-based proteins, as well as resorting to other protein sources (e.g., insect proteins) and to innovative biotechnological processes such as the production of cultured meat (i.e., culturing animal cells in vitro) or, similarly, the production of eggs without chickens or milk without cows. Consequently, in an attempt to meet these needs for protein sources for human nutrition, existing resources have to be properly exploited. Cereals are considered a good source of proteins since their content can vary from approximately 10% in the case of corn to 17% in the case of oat. From this it becomes apparent that the utilization of proteins from cereal by-products is very likely a viable option to support and ensure a well-balanced global protein demand to supply ratio [8]. There are major differences regarding the composition of proteins (type and molecular weight) in the wheat endosperm and outer grain layer, since the first ones contain mainly glutenins and gliadins while albumins and globulins predominate in bran [153]. The higher nutritional value of bran proteins can be attributed to the higher levels of essential amino acids, since these proteins presented therein serve as the main source of amino acids during seed germination. However, cereals are usually deficient in certain amino acids such as lysine [154]. It is important to point out that since the milling process does not generate high temperatures or other detrimental conditions to a level that can cause protein denaturation, bran proteins can be viewed as native high-quality proteins. On the contrary, the proteins in brewer’s spent grains or dried distiller’s grains are degraded, aggregated or even denatured and, in certain cases, can be useful as a source of amino acids or nitrogen (N) [8].

The recovery of proteins from cereal processing by-products has been extensively reviewed recently by Balandran-Quintana [155]. Several extraction methods were described, and it was pinpointed that despite alkaline extraction coupled with isoelectric precipitation has been the most applied technique; there are some concerns about its negative effect on the protein properties. It was highlighted that rice bran as a by-product from cereal industry has received the foremost attention regarding extraction, characterization and proposed applications. Since rice bran proteins are hypoallergenic, they have been proposed for the production of gluten-free products while their hydrolysates have been used in bulk oil emulsions due to their antioxidant activity [156]. A very nutritious flour has been developed from corn oil rich sperm using an alternative method to the alkaline extraction, consisting in drying, aspiration, sieving, lipid extraction and milling. The resulting flour not only contained 30% protein but the essential amino acid balance as well as the protein efficiency ratio was similar to those of caseins [157]. Recently, the effect of three combinations of bioprocessing methods by lactic acid bacteria (LAB) fermentation, cell wall hydrolysing enzymes and phytase on the biochemical and functional properties of wheat bran protein isolates was studied. It was established that the bioprocessing increased the protein (up to 80%) and fat (up to 22.8%) content in the isolates, while additional proteins were identified by the electrophoretic pattern of the protein isolate bioprocessed with enzymes [158].

Despite the fact that several strategies have been employed for improving the extractability of cereal by-product proteins, there are still certain difficulties in their commercialization. Indeed, novel methods for extraction of proteins (or other applications) often face various technical, economic, environmental and legislative considerations. Novel extraction procedures can be energetically high demanding and/or the downstream processing requires further technological developments and improvements. New by-products can be generated with new processes requiring attention to find a viable end-use in a circular economy approach. Moreover, conversion of the processing units is typically difficult to implement in the industry because of natural inertia and existing economic lobbies or simply because some novel advanced technological solutions require substantial initial capital, fixed- and/or variable costs. Finally, new technologies face frequently legislative voids or the need for approval from the regulatory authorities and standardisation bodies. These features represent typical technical, environmental and economic bottlenecks to be overcome before they can turn into energetically efficient, environmentally sustainable and economically feasible technologies and so that they can penetrate into the market.

## 6. Biopolymer-Based Packaging from Cereal By-Products

The synthetic petroleum-based plastics in food packaging systems have raised environmental concerns due to their non-biodegradability nature as well as the depletion of natural unrenewable resources [159]. In this respect, the use of biopolymers as packaging materials is rather promising and has received much attention in the last few decades. Various polymers have been examined in an attempt to improve the barrier, mechanical and thermal properties of the bio-polymer-based packaging films. Under this context, polymers obtained from renewable resources and utilization of agro-industrial waste and by-products are promising alternatives. Nowadays, their use is becoming even more attractive since the scientific advances over the last decade have diminished the costs of converting organic materials into chemicals that can be further supply other markets like the polymer industry [160].

Various natural resources and residues have been used for the production of cellulose nanofibers and nanowhiskers so far [159,161]. More studies are however being conducted to find new alternative sources to obtain nanocelluloses. In this regard, oat husk residues from cereal processing industry were converted into cellulose nanofibers and were used as a reinforcing agent in the production of bio-composites for packaging [162]. Moreover, wheat bran can be used as a source of phenolic and aromatic compounds, acting as key-intermediates for the production of biopolymers. Among all, ferulic and vanillic acids are the most available compounds in plant-based wastes and can be used for the production of natural polyesters [163]. One of the most promising applications though is the production of polylactic acid (PLA) by polycondensation of lactic acid. Various processing techniques can be applied to PLA for the formation of packaging materials (packaging, films and edible coatings) such as injection moulding, extrusion, blow moulding and thermoforming processing [159,164]. PLA packaging films have been thermoformed into trays for packaging of salads, ready-to-eat meals and deli products, among other uses. In comparison with non-renewable hydrocarbon-based polymers, PLA solutions unfolded by life-cycle assessments (LCA) lower product environmental footprints (PEFs), including lower carbon dioxide (CO_2_) emissions. Since large amounts of CO_2_ are absorbed when corn or other crops used for PLA production are cultivated, the use of PLA results in emissions of fewer greenhouse gases compared to the hydrocarbon-based polymers [159,165]. The appropriateness of lactic acid production from cereal-based by-products has already been demonstrated in distillery silages and wastes from bioethanol and beer production [166,167].

Recently, a novel bioplastic has been developed from rice straw, an agricultural waste that generally is not recovered. Interestingly, based on the environmental relative humidity, the developed material exhibited a dual mechanical behaviour that can be further exploited to obtain thin edible films and paper sheets or to drive shape memory effect [168]. In another recent study, wheat lignocellulosic by-products, i.e., straw and bran were used for the co-production of enzymes and bio-based materials, with a possible application as packaging via the compression moulding technique. In general, the straw films were stiffer than the bran derived ones, and the highest Young’s modulus was obtained for the biologically pre-treated bran [169].

The use of rice bran proteins in biodegradable films has also been studied [170,171,172]. However, these films possess inferior mechanical and gas barrier properties when compared to the synthetic ones. Wang et al. [173] evaluated the effect of protein aggregates on the physical, mechanical and thermal properties of the rice bran protein films and it was found that the formation of aggregates can have a positive impact on the produced films, especially at a pH value of 11. Furthermore, the use of AXs or AXOS for packaging applications has been investigated either alone or as part of composites. High molecular mass AXs or mixtures of AXOS and AXs were successfully added to chitosan-based films without affecting their morphology and their gas barrier properties. The incorporation of 0.2% of AXs or AX/AXOS into the chitosan matrix allows obtaining films with added potential functionalities in addition to those of chitosan, namely prebiotic and/or dietary fibre properties of AXOS and/or AXs, respectively [174]. Finally, the topographical and mechanical properties, as well as the biodegradability, of films consisting of AXs from wheat and maize bran and dried distiller’s grains have been assessed, with promising results [175,176].

## 7. Valorisation of Cereal By-Products through New Product Development (NPD)

In our contemporary society, people are becoming more conscious about the importance of a good and balanced nutrition and adoption of a healthy life-style. At the same time, increasing epidemiological evidences have demonstrated that the consumption of sufficient amounts of cereal dietary fibres and phytochemicals could be beneficial against chronic and degenerative diseases [50,177].

The adequate daily total fibre intake (from food origin) recommended by the World Health Organization and the Food and Agriculture Organization is ca. 25–30 g. However, for the Western population the average daily fibre intake is still far below the recommended levels [174]. Moreover, new products, formulations and processes in the baking industry have numerous possibilities to be explored. Examples are the breads suitable for celiac patients, modified texture bread, biofortified bread with vegetable microgreens, leaves, minerals and vitamins, new healthy baked goods and patisserie formulations, ready-to-eat instant porridges, β-carotene-enriched sorghum porridges obtained by co-extruding sorghum flour with orange flesh sweet potato flour, high calorie puree or modified-texture food products. New products and formulations targeted to age groups are also an important market niche, viz. children, pregnant women and nursing mothers, seniors, athletes, people with weakened immune systems or serious autoimmune disorders (e.g., celiac disease), and specific modified-texture foods (e.g., gels) for a population with mastication and swallowing problems.

In that respect, the development of cereal-based products enriched with fibre-rich bran seems to be a very promising approach. Rice bran fractions have been used in order to produce acceptable low-fat bakery products enriched in fibres. Defatted rice bran was successfully used for the production of fibre- and mineral-enriched pan breads. However, the wheat flour commonly used was replaced only by 5% in order not to affect the quality characteristics of the final product [178]. In another study, microwaved stabilised defatted rice bran was used for preparing cookies and it was evidenced that it could be used up to 20% without adverse effects in the quality attributes of the final product [179]. The use of oat, wheat and rye bran in extruded corn snacks has been assessed, and it was found that the selected bran can be applied in the formulations by as much as up to 20% [180]. Along with the enhancement in dietary fibres, the replacement of wheat flour with rice bran in bakery products resulted in a significant increase in the vitamin B group, especially niacin, and minerals such as iron, zinc, phosphorus and potassium [181]. Apart from the changes in the quality attributes of the final product, another drawback for the use of cereal bran in food formulations is its significant content of phytic acid. In a recent study, cereal brans (from rice, rye, wheat and oat) were dephytinized by two different methodologies (phytase enzyme and malt flour) and further used in noodle and pasta formulation (at 20%). The resulted products exhibited low levels of this anti-nutritional compound and, at the same time, increased fibre and phenolic contents [182]. The use of fermented maize milling by-products (25%) in bread making gave also very positive results. Other advantages commonly related to sourdough fermentation, such as the increase in protein digestibility, the decrease in starch hydrolysis and the degradation of phytic acid, were observed [183].

As previously mentioned, wheat bran contains high amounts of dietary fibres but low levels of soluble dietary fibres. Enzymatic processing of cereal bran is related with the conversion of insoluble dietary fibres to soluble ones with the concomitant increase in the beneficial effects [184,185,186]. A combination of different enzymes (i.e., xylanase, β-glucanase, α-amylase and cellulases) was more effective for enzymatic degradation of insoluble dietary fibres than using single enzymes [186]. In order to increase the amount of soluble fibre and improve the quality of baked products, different processes such as extrusion and enzymatic hydrolysis were applied on cereal bran [185]. Wen, Niu, Zhang, Zhao and Xiong [186] used a combination of enzymes with micronization treatment to further modify the structural and functional properties of cereal bran. In addition to enzymes, solid-state fermentations (SSF) of (wheat and oat) bran with microorganisms such as baker’s yeast (*Saccharomyces cerevisiae*) resulted in an increase in phenolic content and antioxidant activity in the bran, revealing the effective impact of fermentation to produce valuable products to include in food recipes [187]. Using probiotics (lactic acid bacteria) improved the functional properties of wheat bran and germ, as well as increasing dietary fibre, protein digestibility, nutritional indexes and lowering the in vivo glycaemic index. Application of sourdough fermentation technology in bread enriched with rice bran resulted in increased loaf volume, phenolic content and consumer acceptance [188]. In another study, incorporation of sourdough from bran in bread making resulted in bread with an appealing aroma and a longer shelf-life [189].

Beta-glucans have different potentials in food applications (i.e., as thickeners, fat replacers, stabilizers, etc.). Incorporation in different proportions (0.5–2%, *w/v*) in full fat yoghurt preparations improves the whey separation (syneresis) during processing, and the viscosity, texture profile and sensory characteristics during storage, resulting in a thicker and a more compact texture [190]. Oat and barley β-glucan applied at concentrations of 1, 2 and 4% reached greater emulsion stability and relatively high values of apparent viscosity, indicating that they act as viscosifier and can be applied in a variety of food products to improve their texture and viscosity [191].

Oat β-glucan isolate was used as a hydrocolloid in order to improve the quality of gluten-free breads [192]. The use of pure β-glucan isolates in breadmaking was associated with increased resistance against deformation, flow ability and elasticity of the doughs under low stress forces. Factors such as the concentration and molecular weight of β-glucans in combination with flour type used, affect the rheological behaviour of baking doughs [193]. Farinograph water absorption, moisture content, water activity and specific volumes of breads increase whereas the firmness decreases with the increase in β-glucan level, as well as its molecular weight [194]. Besides the level and molecular weight of β-glucans, the water added is a key factor that could improve rheological behaviour of β-glucan enriched doughs [195]. Appropriate combinations with added β-glucans can result in improved quality of flours with low breadmaking quality. Processing conditions applied during preparation of foods with high β-glucan content alter the molecular weight, solubility and consequently the viscosity of the food products. Ames et al. [196] stated that heat treatment of cereal grains results in products with high β-glucan viscosity. Depending on the molecular weight and structure, β-glucans can increase the viscosity; modify the rheology, texture and sensory properties of the food products. Modification of β-glucans can be achieved by a number of methods in order to have the desired physicochemical, rheological and functional properties. In general, the physical modification affects only the spatial structure of β-glucans whereas the methods involving chemical agents, enzymes, irradiation and mechanical treatment affect both spatial conformation and primary structures [197].

Rice bran has also been used in muscle food product formulations due to its high antioxidant activity. More specifically, rice bran hydrolysates have shown their effectiveness in reducing fat uptake during frying and controlling lipid oxidation of fried fish cake during frozen storage [198], while substitution of tapioca starch (tapioca or gum is the starch extracted from cassava) in meatballs by 50% of Searng^®^ rice bran doubled the antioxidant activity [199]. Oleogels, gels in which the liquid phase is oil, are a relatively novel and very promising fat replacement technology. In this regard, the impact of replacing pork fat with rice bran wax oleogels in the properties of frankfurter-type sausages was assessed. The very positive results in terms of textural and organoleptic characteristics point to the potential use of another compound recovered from cereal by-products [200].

Finally, cereal by-products are also gaining attention for their use in non-food related industries. Compounds such as rice husk ash have been evaluated for the production of green and sustainable concrete in an attempt to minimize the significant amount of carbon dioxide (CO_2_) emissions commonly released during concrete production [201].

**Figure 4:**
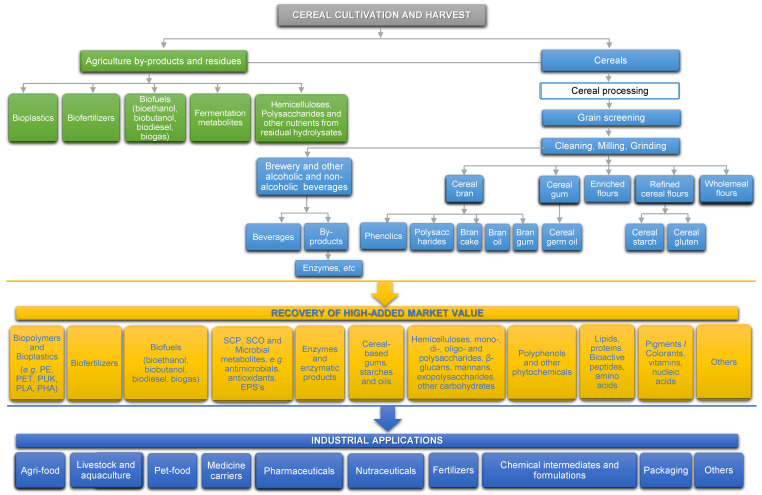
Valorisation of by-products and wastes from cereal-based processing industry.

The overall approach and considerations given in this review article based on its aims are schematically summarized in the following Figure 4.

## 8. Environmental and Economic Sustainability Outlook

The ever-growing amounts of cereal-based processing by-products lead to the imminent need to develop more sustainable and eco-friendly processing technologies as well as in exploring innovative solutions for its employment in a circular economy approach. The extraction of high-added functional compounds and fractions from these materials can be employed in various industrial sectors and offers true alternatives to synthetic and non-renewable materials. Such efforts also happen in a context of an increasing trend of the global market, and the perceived new values and concerns from our society. Yet, it also reinforces the goals of the “*2030 Agenda for Sustainable Development*” from the United Nations in striving for the promotion of sustainability with rational use of edible resources [3]. Taking into consideration the fact that plant-based diets are the most beneficial for the human health and for the environmental sustainability of our Planet, exploration of food and feed formulations, based on edible fibres, proteins and other compounds originated from cereal-based processing by-products, effectively represents a scientific and technological (S&T) hotspot and simultaneously brings great challenges to be overcome.

Although cereal-based by-products can be an excellent resource for such a diversified number of compounds with high-added values, the operation costs and efficiency are, as previously mentioned, major economic and technical bottlenecks to be overcome through the development of novel and increasingly efficient biotechnological systems. Indeed, the costs associated with conventional recycle and reuse solutions are frequently higher than simply resorting to the landfill or composting (in a strictly immediate economic point of view). In addition, conventional extraction and recovery procedures are many times more environmentally impacting (i.e., with higher environmental footprints) than those based on conventional solutions. In this context, it is required to explore new insights and develop deeper scientific-technological efforts to efficiently valorise cereal-based by-products and residues. This review opens several windows of opportunities based on the constituents presented therein, on promising available biotechnological tools and the always-important future perspectives.

Particularly, it is still necessary to develop and optimize environmental-friendly extraction, separation and purification techniques and methodologies, so as to attain process intensification (including energy and water consumption reduction). Additionally, integration of technologies (for example, combining pre-treatments with bioconversion or, instead, combining chemical, physical, microbial and enzymatic processes), efficient and economically feasible scale-ups, and finding adequate channels to reintroduce innovative biotechnological solutions into the market are required. Actual EU policies are just coming in this way. Overall, this is all about the deconstruction of cereal-based food and feed by-products and residues and further incorporates the resulting bulk fractions or isolated purified compounds into new industrial chains. The main challenge is, however, to develop reliable biotechnological processes and systems with increased cost-effectiveness, and able to be easily constructed and implemented, scalable and operationalised, while offering easily and efficiently large-scale outputs to reach substantial market dimensions. Thus, the attention of the researches and technicians must continue focused on these paradigms. In fact, economically feasible processes can only be achieved within high-value market products and production at a large scale. Economic feasibility can be achieved with increased competitiveness obtained through the development and optimization of more efficient, sustainable and cost-effective downstream processes and lower initial capital costs.

The concerns and proposals found in this review article are in agreement with the recent EU strategies and policies [4,5,7] for food production, processing, distribution, consumption, prevention of food loss and waste and reuse, i.e., throughout the entire food and feed product life cycles, “*from cradle to grave*”. Only with these strategies, based on an efficient circular economy [4], will it be possible to mitigate climate change; restore its biodiversity and save our Planet for future generations; ensure robust and resilient food and feed systems with neutral/positive environmental impacts; and boost food quality and safety, as well as nutrition and public health and, above all, guarantee zero tolerance to hunger in the World.

## 9. Conclusions

Besides the environmental and economic aspects, discarded cereal- and pseudocereal-based by-products represent an important loss of valuable biomass and nutrients (as already shown in Figure 4), including many biochemical compounds such as edible fibres, hemicelluloses, β-glucans, resistant starch, arabinoxylans, mono-, di-, oligo- and polysaccharides and other carbohydrates, proteins, bioactive peptides, amino acids and enzymes, lipids (mono- and polyunsaturated fatty acids in free forms, acylglycerols and sterol esters, stanols, glycolipids and phospholipids), polyphenols (phenolic acids, gallotannins, procyanidins, etc.) and other bioactive phytochemicals, vitamins, minerals and other micronutrients, nucleic acids, etc. Cereal-based by-products can be converted as well into biopolymers and bioplastics, biofertilizers and biofuels (bioethanol, biodiesel, bio-butanol and biogas). Their application in fermentative processes can lead to the production of single cell proteins and oils, enzymes and bioactive microbial metabolites.

Cereal-based by-products can be an important source of enzymes with industrial interest, such as glycoside hydrolases, laccases, oxidoreductases, lipoxygenases, lipases, phytases, proteases, α-amylases, hemicellulases, proteases, esterases, decarboxylases, reductases and transglutaminases. Likewise, numerous microbial metabolites from bacteria, yeasts and moulds (filamentous fungi) can be produced, through the fermentation of cereal by-products, with antioxidant, antibacterial and antifungal activities, e.g., a long list of organic acids (such as lactic, acetic, propionic, formic, succinic, propionic, butyric, γ-aminobutyric, sorbic, fumaric and benzoic acids), hydrogen peroxide (H_2_O_2_), CO_2_, ethanol and other alcohols, diacetyl (butanedione or butane-2,3-dione), aldehydes and other volatile compounds, esters and carbonyls, bacteriocins and bacteriocin-like inhibitory substances (BLIS), small peptides and amino acids and exopolysaccharides (e.g., β-glucans, levans, fructans and fructo- and glucooligosaccharides).

Conversely, the hydrolysis of lignocellulosic or starch residues from cereal-based by-products can yield fermentable sugars to be further fermented and converted into biofuels, enzymes, bioplastics, β-glucans, mannans, etc. Residual biomass can feed further employed in anaerobic digestions, to produce renewable bioenergy and/or biofertilizers, or undergo pyrolysis processes. Lignocellulosic residues can also be used in microbial solid-state fermentations. Bacterial fermentation of cellulose can yield the high-added value 2,3-butanediol and bio-butanol. The fermented cakes can also be used in pet-food formulations. Furthermore, cereal-based by-products can be applied in the design of bio-based plastics, for example bio-based polyethylene (bio-PE), bio-based polyethylene terephthalate (bio-PET), bio-based polyurethane (bio-PUR), polylactic acid (PLA), modified starch, cellulose derivatives and polyhydroxyalkanoates (PHA). All the above compounds and fractions possess high-added values and a transversal application in the industry, following an approach of circular economy and minimum or zero residues generation, viz.: food and feed, pet-food, medicine, pharmaceutical, nutraceutical, fertilizers, chemical intermediates and packaging.

This review was intended to give useful information concerning the main high-added value compounds found in cereal-based by-products and the wide range of technologies used for their isolation as well as their unlimited industrial applications.

## Figures and Tables

**Table 1:** Different techniques reported in recent published literature (2016–2020) to improve extraction of arabinoxylans and β-glucans from cereals.

Compound	Techniques	References
**Arabinoxylans**	Fermentation	[102,103,104]
	Ultrasonic/microwave	[105,106]
	Enzymatic treatment	[107,108,109,110]
	Milling fractions	[111,112,113]
	Alkaline treatment	[114,115,116,117]
	Nixtamalization	[118]
	Starch removal	[119]
	Extrusion	[80,86,89,120]
	Subcritical water treatment	[119,121]
**β-glucans**	Ultrasound	[122]
	Roller-milling/pearling	[123]
	Milling	[124,125,126]
	Fermentation	[127,128]
	Enzymatic treatment	[129,130]
	Extrusion	[131,132,133,134]
	Subcritical water treatment	[119,135,136]

**Table 2:** Recent advances for the extraction of phenolic compounds from various by-products.

Extraction Method	Conditions	Cereal By-Product	Reference
Supercritical carbon dioxide	Pressure (15–35 MPa), temperature (40–60 °C) and CO_2_ + ethanol (0–60% ethanol concentration, *v/v*)	Brewer’s spent grain	[139]
	Temperature (40 and 60 °C), pressure (30 and 40 MPa) and ethanol (0, 5 and 10%)	Rice bran	[140]
Green method using glycerol	Temperature (40–70 °C), concentration of glycerol (10–70%, *v/v*) and liquid-to-solid ratio (10–40 mL g^−1^)	Rice bran	[141]
Solvent extraction	Absolute methanol, 80% methanol, absolute ethanol, 80% ethanol, and 50% acetone	Wheat bran and barley husk	[142]
Thermal processing coupled with ultrasound extraction	Temperature 80 °C for 10 min	Wheat and oat bran	[143]
Pressurized solutions	Concentrations of ethanol in water (0–80%), temperature (130–160 °C) and extraction times (20–60 min)	Wheat bran	[144]
Ultrasonic-assisted extraction (UAE)	Ethanol and limonene, temperature 51 and 60 °C respectively	Rice berry bran	[145]
	Temperature (30–60 °C), pH (2–4), solvent concentration 920–60%), time (10–60 min)	Black and purple rice bran	[146]
	Time (10–30 min), ethanol concentration (30–70%), solvent to solids (mL g^−1^)	Red sorghum bran	[147]
	Temperature (20–90 °C), time (1–25 min)	Defatted oat (*Avena sativa* L.) bran	[122]
Steam explosion-assisted extraction	Temperature 215 °C for 120 s	Wheat bran	[148]

## Abbreviations

Araf	α-L-arabinofuranosyl
A/X	Arabinose to xylose ratio
AXOS	Arabinoxylan-oligosaccharides
AXs	Arabinoxylans
BLIS	Bacteriocin-like inhibitory substances
bio-PE	Bio-based polyethylene
bio-PET	Bio-based polyethylene terephthalate
bio-PUR	Bio-based polyurethane
BMI	Body mass index
C	Carbon
CO_2_	Carbon dioxide
CVD	Cardiovascular disease
DP	Degree of polymerization
DAG	Diacylglycerols
DDGS	Dried distiller’s grain with solubles
d.b.	Dry basis
EFSA	European Food Safety Authority
EU	European Union
EPS	Exopolysaccharides
FA	Fatty acid
FA	Ferulic acid
FAO	Food and Agriculture Organization
FDA	Food and Drug Administration
GABA	γ-aminobutyric acid
GI	Glycaemic index
H_2_O_2_	Hydrogen peroxide
LAB	Lactic acid bacteria
LCA	Life cycle assessment
LDL	Low density lipoproteins
MAG	Monoglycerides
MUFA	Monounsaturated fatty acids
NPD	New Product Development
O_2_	Molecular oxygen
N	Nitrogen
O	Oxygen
PHA	Polyhydroxyalkanoates
PLA	Polylactic acid
AX	Polysaccharide arabinoxylan
PUFA	Polyunsaturated fatty acids
PEF	Product environmental footprint
S&T	Scientific-technological
SCO	Single cell oils
SCP	Single cell proteins
SSF	Solid-state fermentation
SE	Sterol esters
SWE	Subcritical water extraction
SC-CO_2_	Supercritical carbon dioxide extraction
TAG	Triglycerides
UAE	Ultrasonic(/Ultrasound)-assisted extraction
UN	United Nations
WEAX	Water-extractable arabinoxylans
WUAX	Water-unextractable arabinoxylans
WHO	World Health Organization
